# Proteogenomic Analysis of *Epibacterium Mobile* BBCC367, a Relevant Marine Bacterium Isolated From the South Pacific Ocean

**DOI:** 10.3389/fmicb.2018.03125

**Published:** 2018-12-21

**Authors:** Sabine Matallana-Surget, Johannes Werner, Ruddy Wattiez, Karine Lebaron, Laurent Intertaglia, Callum Regan, James Morris, Hanno Teeling, Manuel Ferrer, Peter N. Golyshin, Dimitrios Gerogiorgis, Simon I. Reilly, Philippe Lebaron

**Affiliations:** ^1^Division of Biological and Environmental Sciences, Faculty of Natural Sciences, University of Stirling, Stirling, United Kingdom; ^2^Department of Biological Oceanography, Leibniz Institute of Baltic Sea Research, Rostock, Germany; ^3^Department of Proteomics and Microbiology, Interdisciplinary Mass Spectrometry Center (CISMa), University of Mons, Mons, Belgium; ^4^Sorbonne Universites, UPMC Univ Paris 06, CNRS, Laboratoire de Biodiversité et Biotechnologies Microbiennes (LBBM), Observatoire Océanologique, Banyuls/Mer, France; ^5^Sorbonne Universites, UPMC Univ Paris 06, CNRS, Observatoire Océanologique de Banyuls (OOB), Banyuls/Mer, France; ^6^Department of Molecular Ecology, Max Planck Institute for Marine Microbiology, Bremen, Germany; ^7^Department of Applied Biocatalysis, Institute of Catalysis, CSIC, Madrid, Spain; ^8^School of Natural Sciences, University of Bangor, Bangor, United Kingdom; ^9^Institute for Materials and Processes, School of Engineering, University of Edinburgh, The King's Buildings, Edinburgh, United Kingdom

**Keywords:** *Epibacterium mobile*, proteogenomic, *roseobacter*, stress response and adaptation, quantitative proteomics

## Abstract

*Epibacterium mobile* BBCC367 is a marine bacterium that is common in coastal areas. It belongs to the *Roseobacter* clade, a widespread group in pelagic marine ecosystems. Species of the *Roseobacter* clade are regularly used as models to understand the evolution and physiological adaptability of generalist bacteria. *E. mobile* BBCC367 comprises two chromosomes and two plasmids. We used gel-free shotgun proteomics to assess its protein expression under 16 different conditions, including stress factors such as elevated temperature, nutrient limitation, high metal concentration, and UVB exposure. Comparison of the different conditions allowed us not only to retrieve almost 70% of the predicted proteins, but also to define three main protein assemblages: 584 essential core proteins, 2,144 facultative accessory proteins and 355 specific unique proteins. While the core proteome mainly exhibited proteins involved in essential functions to sustain life such as DNA, amino acids, carbohydrates, cofactors, vitamins and lipids metabolisms, the accessory and unique proteomes revealed a more specific adaptation with the expression of stress-related proteins, such as DNA repair proteins (accessory proteome), transcription regulators and a significant predominance of transporters (unique proteome). Our study provides insights into how *E. mobile* BBCC367 adapts to environmental changes and copes with diverse stresses.

## Introduction

With oceans covering ~70% of the planet's surface they represent the largest habitat on Earth. Living biomass in the oceans is dominated by microorganisms (Church, 2009; DeVries et al., 2017). In contrast to blue water open oceans, coastal marine environments represent more heterogeneous habitats that provide a wider spectrum of accessible dissolved organic matter. These conditions favor copiotrophic generalist bacteria over specialists (Lauro et al., 2009). These generalists are characterized by large pools of catabolic and transporters as well as stress response functions, which enable them to profit from the ample nutrient supply and to cope with changing environmental conditions in their coastal ecosystems (Lauro et al., 2009; Christie-Oleza et al., 2012).

*Epibacterium mobile* BBCC367, formerly known as *Ruegeria mobilis* BBCC367 (Wirth and Whitman, 2018), a member of the *Roseobacter* clade (Lee et al., 2012), is a key component of marine bacterioplankton, as 15% of bacterial cells in the open ocean and 20% in coastal waters are members of this group from tropical to polar regions (Moran et al., 2007; Gram et al., 2010). Members of the *Roseobacter* clade feature diverse metabolic capabilities that foster their widespread abundance, particularly in temperate and deep pelagic oceans (Luo and Moran, 2014).

**Figure 1:**
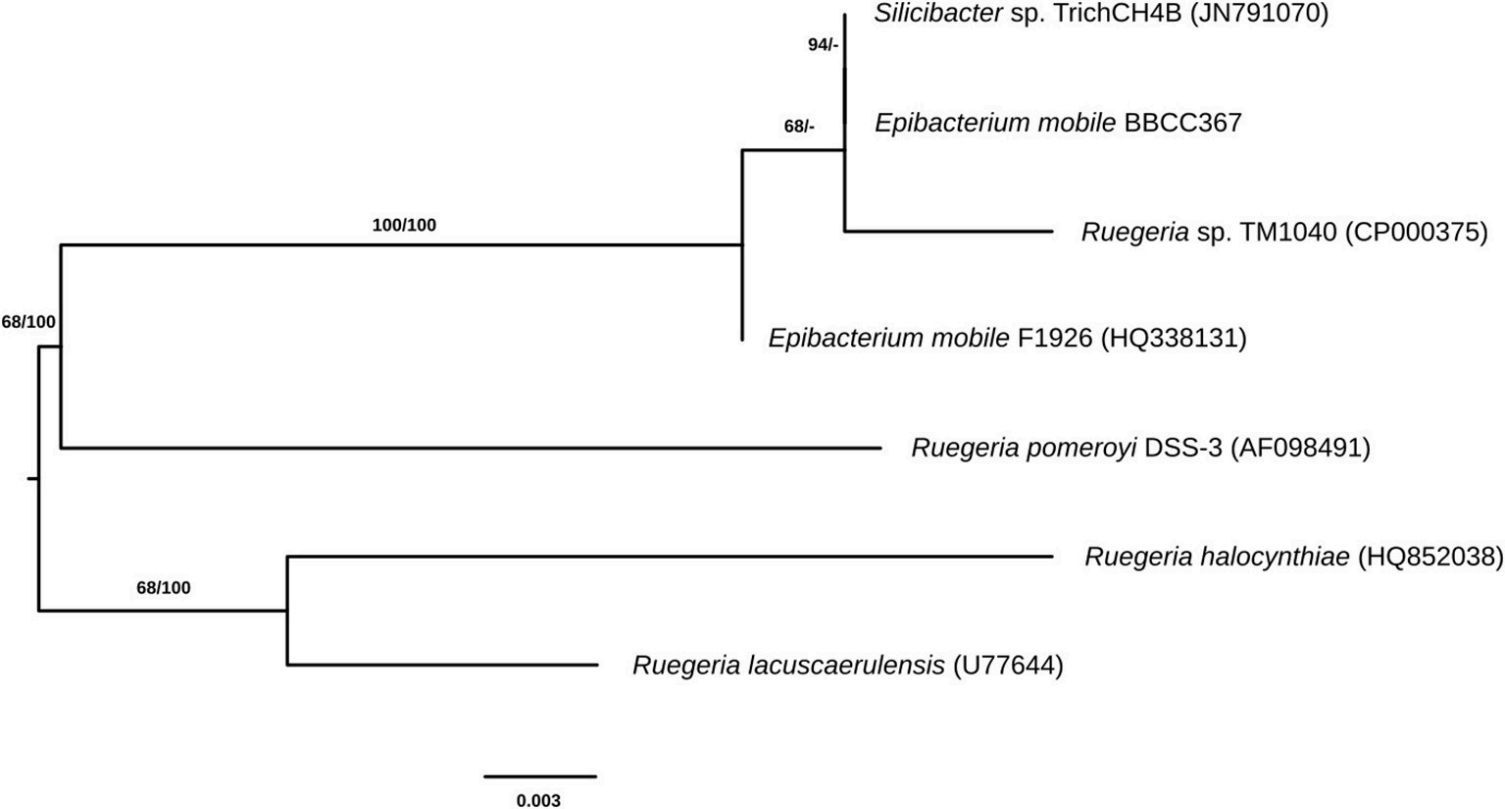
Phylogenetic tree of closely related species to *E. mobile* (the identifiers in brackets are IDs from the ribosomal database of full-length 16S sequences).

*E. mobile* BBCC367 is a dark-brown pigmented, facultative aerobic bacterium (Lee et al., 2007) that was isolated from the South Pacific Ocean, off the coast of Chile in 2004 (Claustre et al., 2008; Matallana-Surget et al., 2012b). *E. mobile* BBCC367 is closely related to *Ruegeria pomeroyi* (formerly *Silicibacter pomeroyi*, Figure 1) and both species share similar geographical distributions (Sonnenschein et al., 2017). *R. pomeroyi* was the first member of the *Roseobacter* clade to have its genome completely sequenced and annotated, revealing a large pool of genes with roles in adaptations to changing environmental conditions (Moran et al., 2007). Nevertheless, little is known about the fraction of expressed proteins that is continuously expressed for maintaining housekeeping functions and the tightly regulated fraction that is expressed only in response to environmental changes (Christie-Oleza et al., 2012).

Marine bacteria near the ocean surface are particularly impacted by solar radiation. *E. mobile* BBCC367 showed a high resistance to UVB exposure (Matallana-Surget et al., 2012b) and thus, was selected as a model organism to study UV resistance. Most bacterial species when exposed to elevated levels of solar UV radiation (UVR) respond with decreases in abundance as well as their amino acid uptake, exo-enzymatic activities, oxygen consumption, protein, and DNA synthesis (Alonso-Saez et al., 2006; Matallana-Surget and Wattiez, 2013). Nonetheless, Farías et al. (2009) showed *Roseobacter* abundances to increase with solar exposure, even at very high altitudes observed in the Laguna Vilama, a hypersaline Andean lake in Chile (4,650 m) exposed to high doses of UVR. Within the two dominant subgroups of *Alphaproteobacteria* in the Mediterranean sea, bacteria belonging to the *Roseobacter* clade showed higher resistance than members of the SAR11 cluster (Alonso-Saez et al., 2006). UVR resistance is often attributed to a low GC content, which limits cytosine-containing photoproduct formation (Agogué et al., 2005; Matallana-Surget et al., 2009). However, *E. mobile* BBCC367 has a DNA G+C content of 58 mol%, which makes the high UV tolerance in *E. mobile* BBCC367 even more remarkable.

Proteogenomics is the integrated study of proteomics and genomics with the aim to obtain the complete resolution of a species proteome (Armengaud, 2012). In our study, we sequenced and annotated the genome of *E. mobile* BBCC367 and used high-throughput proteomics to assess its proteome. We cultivated *E. mobile* BBCC367 under 16 different conditions to access as many non-redundant proteins as possible and to increase coverage of the theoretical proteome. *E. mobile* BBCC367 was cultivated under different temperatures (4°C, 40°C), oxic vs. anoxic conditions, in presence of different metals (copper, nickel, zinc, cobalt), under different times of UVB exposure, and harvested at different physiological states (exponential phase, late stationary phase, very late stationary phase). The overall aim of this study was the investigation of *E. mobile* BBCC367's proteome with a particular focus on its resistance to diverse forms of stress, in particular UVB radiation.

## Materials and Methods

### Bacterial Strains and Culture Conditions

*E. mobile* BBCC367 was isolated during the 2004 BIOSOPE cruise from the coastal waters off Chile (Claustre et al., 2008). *E. mobile* BBCC367 is maintained in the Banyuls Bacterial Culture Collection or BBCC (https://collection.obs-banyuls.fr/catalogue.php). Pre-cultures were grown aerobically on a rotary shaker (120 rpm) at 25°C in either marine broth (MB) for the proteogenomic study or in artificial sea water (250 mL) with 3 mM D-glucose (ASW-G), vitamins and trace elements (Eguchi et al., 1996) for the quantitative proteomics study. *E. mobile* BBCC367 was cultured under 16 conditions to obtain the highest proteome coverage. For proteogenomics (conditions 1 to 16, Table 1), cells were cultivated in MB. In this first study, no biological replicate was performed for each condition. For the 16 conditions, cells were pelleted by centrifugation at 8,000 g for 15 min and the pellets were washed using phosphate buffer saline to remove proteins derived from the MB medium. Label-free quantitative proteomics allowed assessment of the impact of UVB. Experiments were carried out for UVB treatments and dark controls in triplicates. For quantitative proteomics cells were pelleted by centrifugation at 8,000 *g* for 15 min and were stored at −80°C until further use.

**Table 1:** Growth conditions used for proteogenomics and quantitative proteomics.

**Condition**	**Number of replicates**	**Medium**	**Growth condition**	**Growth phase**
1	1	MB	4°C	Exponential phase
2	1	MB	Cold Shock	Exponential phase
3	1	MB	40°C	Exponential phase
4	1	MB	Heat shock	Exponential phase
5	1	MB	Anaerobic	Early stationary phase^*^
6	1	MB	LSP	Late stationary phase^**^
7	1	MB	vLSP	Very Late stationary phase^**^
8	1	SW	Filtered sea water	Early exponential phase
9	1	MB	T0_MET (control condition)	Early stationary phase
10	1	MB	Cu, 1 mM	Early stationary phase
11	1	MB	Cu, 5 mM	Early stationary phase
12	1	MB	Zn, 1 mM	Early stationary phase
13	1	MB	Co, 1 mM	Early stationary phase
14	1	MB	Ni, 1 mM	Early stationary phase
15	1	MB diluted sea salts	UVB 5 h	Early stationary phase
16	1	MB diluted sea salts	Dark 5 h	Early stationary phase
17	3	ASW	UVB 2 h	Exponential phase
18	3	ASW	DK 2 h	Exponential phase

*MB: Marine broth, ASW: Artificial Sea Water*,

* *Early stationary phase (when culture reached peak growth)*,

** *Late and very stationary phase (4 and 10 days, respectively, after the culture reached stationary phase). Gray lines correspond to samples for quantitative proteomics*.

### DNA Extraction and Sequencing

For DNA extraction, the pellet of a fresh culture (50 ml, 25°C, 2 days at 100 RPM) of the strain *E. mobile* BBCC367 was suspended in 9.5 ml TE (10 mM Tris, 1 mM EDTA) buffer. 0.5 ml of 10% SDS, 5 μl RNase A 10 mg/ml and 50 μl of 20 mg/ml of proteinase K were added, mixed thoroughly and incubated 1 h at 37°C. Then, 1.8 ml of 5 M NaCl was added and mixed thoroughly. 1.5 ml CTAB/NaCl solution was added, mixed thoroughly and incubated 20 min at 65°C. An equal volume of chloroform/isoamyl alcohol 24:1 solution was added to the previous solution and after 10 min at 6,000 g at room temperature, the supernatant was transferred into a 0.6 vol of isopropanol until the DNA appeared. The DNA string was hooked with the end of a Pasteur pipette and transfer into a 70% ethanol solution until further analysis.

*De novo* sequencing data production for *E. mobile* BBCC367 was conducted at the Liverpool University Genome Centre on a 454 FLX Ti (454 Life Sciences, Branford, CT, USA) using a standard library (ca. 16x coverage). In parallel, a library sequencing using Illumina HiSeq1500 was done at Fidelity Systems Ltd. (Gaithersburg, MD, USA) with short paired-end 400 bp, average read length of 100 bp and 256x coverage. Genome assembly and gap closure were performed by Fidelity Systems using Phred/Phrap and Consed for the final sequence assembly (Ewing and Green, 1998b; Ewing et al., 1998a; Gordon, 2003). DupFinisher (Han and Chain, 2006) was used for the correction of repeat mis-assemblies. For the full closure, a number of direct sequencing was conducted (Malykh et al., 2004).

### Genome Annotation

Prediction of genes and functional annotation were carried out through the Rapid Annotation using Subsystem Technology (RAST) server (Aziz et al., 2008). Resulting annotations were subsequently imported into a local installation of the GenDB v.2.2 annotation system (Meyer et al., 2003) for data mining, using similarity searches against the NCBI non-redundant protein (Pruitt et al., 2012), InterPro (Hunter et al., 2012), PFAM (Sonnhammer et al., 1997), KEGG (Ogata et al., 1999), COG (Tatusov et al., 2003) databases, as well as predictions of signal peptides with SignalP v3.0 (Nielsen et al., 1997) and transmembrane regions with TMHMM v2.0c (Krogh et al., 2001). Annotations of selected genes were manually curated using JCOAST (Richter et al., 2008). The annotated genome sequence of *E. mobile* BBCC367 was submitted to ENA (LR027553-LR027556).

### Protein Extraction and Quantification

For protein extraction, the thawed cell pellet was re-suspended in one pellet volume of lysis buffer (6 M guanidine chloride), and cells were mechanically broken by sonication on ice (5 cycles of 1 min with tubes on ice, amplitude 30%, 0.5 pulse rate). Broken cells were centrifuged at 16,000 g at 4°C for 15 min. Protein samples were reduced with 25 mM dithiothreitol (DTT) at 56°C for 30 min and alkylated with 50 mM iodoacetamide at room temperature for 30 min. Proteins were precipitated with cold acetone overnight at −80°C, with an acetone/aqueous protein solution ratio of 4:1. The protein pellet was dissolved in 100 mM phosphate buffer (pH 8) containing 2 M urea. Total protein concentration was determined by a Bradford assay. For LC-MS/MS analysis, a trypsic digestion (sequencing grade modified trypsin, Promega) was performed overnight at 37°C, with an enzyme/substrate ratio of 1:25.

### Liquid Chromatography Tandem Mass Spectrometry (LC-MS/MS)

Protein identification and quantification were conducted following a label-free strategy on an ultra-high-performance liquid chromatography–high-resolution tandem mass spectroscopy (UHPLC-HRMS/MS) platform (Eksigent 2D Ultra and AB Sciex TripleTOF 5600 system). Peptides were separated on a 25 cm C18 column (Acclaim pepmap 100, 3 μm, Dionex/LC Packings, Amsterdam, the Netherlands) by a linear acetonitrile gradient [5–35% (v/v), in 15 or 120 min] in water containing 0.1% (v/v) formic acid at a flow rate of 300 nL min^−1^. The instrument was operated in DDA data-dependent acquisition (DDA) mode and MS/MS were acquired across 100–1,800 m/z. A long run procedure was used to acquire quantitative data, and a duty cycle of 3 s per cycle was used to ensure that high quality extracted ion chromatograms (XIC) could be obtained. Protein searches were performed against *E. mobile* BBCC367 genome using ProteinPilot v4.1. Search parameters included differential amino acid mass shifts for oxidized methionine (+15.9949 Da). The identification of the overall set of proteins was validated by manual inspection of the MS/MS ion spectra, ensuring that a series of consecutives sequence-specific b- and y-type ions was observed. For quantification, the quant application of PeakView was used to calculate XIC for all peptides identified with a confidence >0.99 using ProteinPilot.

Quantified proteins were kept with a *p* < 0.05. The false discovery rate (FDR) was calculated at the peptide level for all experimental runs using the decoy option in Mascot; this rate was estimated to be lower than 1% using the identity threshold as the scoring threshold system. The cut-off for a significantly differential regulation was used for a protein showing average increase in abundance above 2 or below 0.5 fold change in the UVB radiation samples relative to their controls.

### Phylogenetic Analysis

Pairwise sequence similarities and phylogenies of the complete 16S rRNA genes of *E. mobile* BBCC367 and six further genomes among the genera *Epibacterium, Ruegeria*, and *Silicibacter* were calculated by the GGDC web server (http://ggdc.dsmz.de/; Meier-Kolthoff et al., 2013). A multiple sequence alignment was created with MUSCLE (Edgar, 2004), maximum likelihood (ML) and maximum parsimony (MP) trees were inferred from the alignment with RAxML (Stamatakis, 2014) and TNT (Goloboff et al., 2008), respectively. For ML, rapid bootstrapping in conjunction with the autoMRE bootstopping criterion (Pattengale et al., 2010) and subsequent search for the best tree was used; for MP, 1000 bootstrapping replicates were used in conjunction with tree-bisection-and-reconnection branch swapping and ten random sequence addition replicates. The sequences were checked for a compositional bias using the X^2^ test as implemented in PAUP (Swofford, 2002).

### Protein Sequences Cluster Analysis

Proteins that behaved similarly across a set of experiments, or with similar abundance profiles were grouped together. Dendrogram construction was performed by means of complete linkage hierarchical clustering, using the “farthest neighbor” distance metric (Murtagh and Contreras, 2011; Khachumov, 2012). The oncoprint was generated using R v 3.5.1 and the ComplexHeatmap package v. 1.17.1 (Gu et al., 2016).

## Results and Discussion

### Theoretical vs. Expressed Proteome

The genome sequence of *E. mobile* BBCC367 consists of 4,712,067 bp, including 4,557 coding sequences (CDS) and 75 RNAs (15 rRNAs and 60 tRNAs). *E. mobile* BBCC367 comprises two chromosomes (3,073 and 1,167 CDS) and two plasmids (125 and 192 CDS) (Table 2). It is noteworthy that second chromosomes are not common among members of the *Rhodobacteraceae* family, and were observed in only 6 out of 74 completely sequenced genomes namely: *Paracoccus denitrificans* PD1222, three strains of *Rhodobacter sphaeroides* (2.4.1, ATCC_17029 and KD131), *Yangia pacifica* YSBP01 and *Yangia sp*. CBB-11M3. The theoretical proteome comprises the complete set of predicted proteins (Matallana-Surget et al., 2009). The expressed proteome represents the complete set of proteins identified by mass spectrometry in the 16 conditions presented in Table 1. One of the main limitations in proteogenomics often relates to the low theoretical proteome coverage. This issue can be overcome by investigating at multiple growth conditions together with several separation and/or fractionation techniques prior to MS/MS analysis (Kucharova and Wiker, 2014). While there are many different techniques to fractionate the proteome all producing different results (Castellana and Bafna, 2010), our experimental design using 16 different growth conditions enabled us to successfully obtain a coverage of 67.6% using only biological fractionation (Table 2). This represents a high coverage compared to other studies, e.g., that of *R. pomeroyi* where 46% of its theoretical proteome was found to be expressed (Christie-Oleza et al., 2012). Chromosomal and plasmid coding genes comprised 94% and 6% of the expressed *E. mobile* BBCC367 proteome, respectively.

**Table 2:** Comparison of the theoretical (coding sequences) and expressed proteome.

	**Theoretical proteome**	**Expressed proteome**	**%Coverage**
Chromosome 1	3,073	2,227	72.5
Chromosome 2	1,167	686	58.7
Plasmid 1	125	70	56.0
Plasmid 2	192	100	52.0
Total	4,557	3,083	67.6

**Figure 2:**
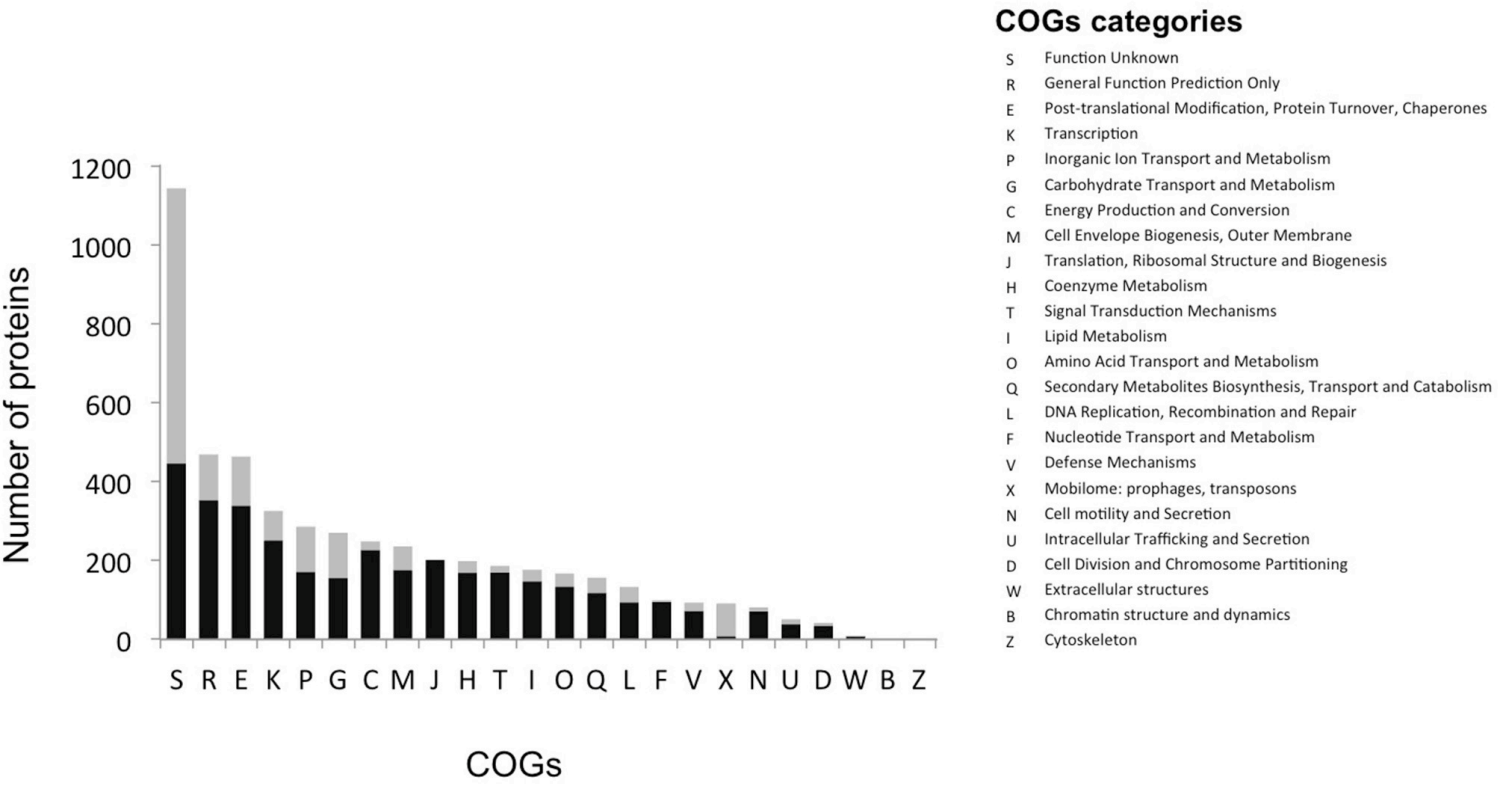
Distribution of proteins into COG categories of the theoretical (gray) and the expressed proteome (black).

**Figure 3:**
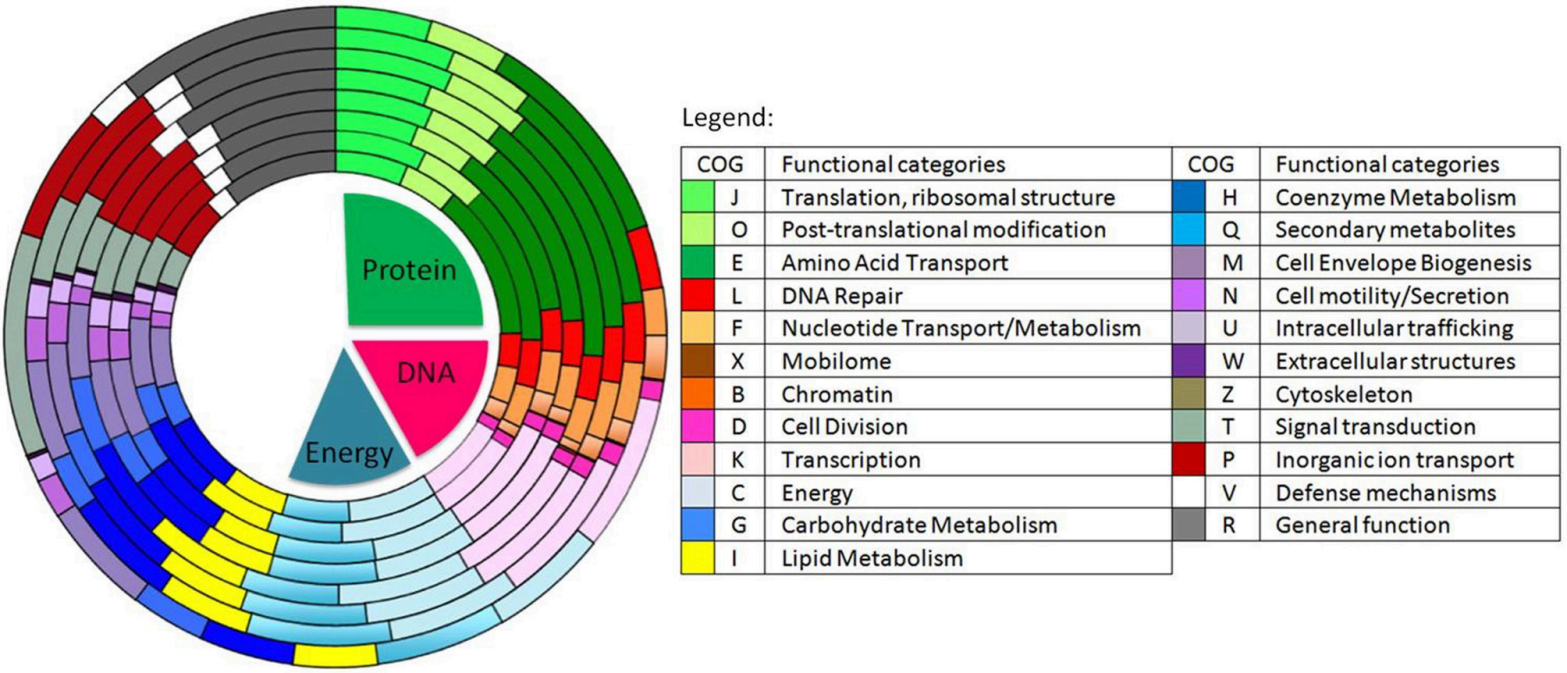
Distribution of the predicted genes into COGs in 8 different species of *Ruegeria* and *Epibacterium*. From the innermost to the outermost circle: *R. halocynthiae, R. lacuscaerulensis, E. mobile* F1926, *R. pomeroyi* DSS-3, *R. TM1040, R. trichCH4B* and our strain *E. mobile BBCC367*. Shades of green represent predicted genes involved in protein functions (e.g., amino acid transport), shades of red to purple represent predicted genes involved in DNA processes (e.g., DNA repair) and shades of light blue to yellow represent predicted genes involved in energy metabolism processes.

The most abundant proteins identified in the theoretical proteome were found in COG S (unknown function) and COG R (general function) with 26 and 11%, respectively, with a small portion of COG S characterized in the expressed proteome (Figure 2). The two largest contributors of the expressed proteome were also found to belong to COGs S and R. The most abundant COGs with a related known function and representing about 1/4 of the genome were found to be involved in proteins metabolism including translation (COG J), protein turnover (proteases, chaperones) (COG O) and amino-acid synthesis (COG E) (Figures 2, 3, green parts). COG J (translation, ribosomal structure and biogenesis) presented a 100% coverage value where all 200 proteins were identified from the theoretical proteome. COG J is ubiquitous and expressed predominantly as these proteins form the universal core of life (Tatusov et al., 1997). In a proteomic analysis of *R. pomeroyi* it was also found that proteins related to COG J (translation, ribosomal structure and biogenesis) (16%) and COG E (amino acid transport and metabolism) (14%) were among the most abundant proteins detected (Christie-Oleza et al., 2012). Moreover, COG E (amino acid transport) represents the largest COG category in 7 different *Epibacterium, Ruegeria*, and *Silicibacter* species (Figure 3), formerly all belonging to *Ruegeria* (Figure 1, Wirth and Whitman, 2018). The second main COGs gathered proteins involved in DNA metabolism including transcription (COG K), DNA repair (COG L), nucleotide metabolism (COG F), and cell division (COG D) (Figures 2, 3, red parts). Finally the third group of proteins was involved in energy production (Figure 2: COGs C and G and Figure 3, blue part).

Genomic comparison of 7 different strains belonging to *Epibacterium, Ruegeria*, and *Silicibacter* (Figure 1) revealed that the most distinct strain was *E. mobile* BBCC367 (external circle, Figure 3). Predicted genes involved in protein and DNA metabolisms and energy presented a comparable distribution between all strains. Both COG R (general function only) and COG T (signal transduction mechanism) were found to be overrepresented in *E. mobile* BBCC367 (Figure 3). Signal transduction occurs when an extracellular signaling molecule activates a specific receptor; generating responses such as changes in enzyme activity or gene expression. Thus, *E. mobile* BBCC367 might be more adapted to respond to environmental variations.

### Theoretical Proteome—Diversity of Metabolic Pathways

#### Diversity of Transporters

**Figure 4:**
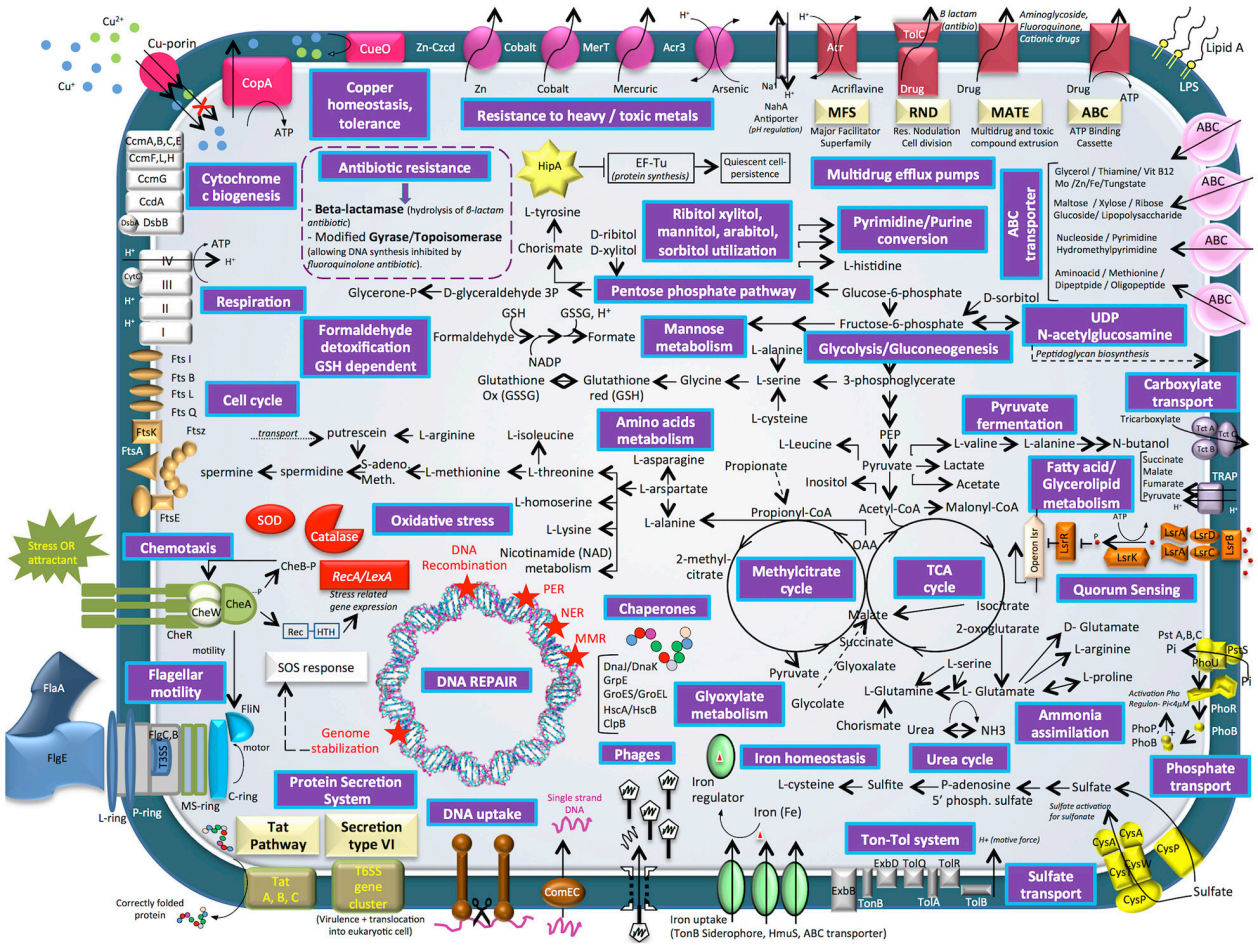
Cell diagram showing the main metabolism pathways of *E. mobile* BBCC367 obtained from the theoretical proteome.

Four main categories of transporters were characterized, i.e., ABC (ATP-binding cassette) transporters, TRAP (tripartite ATP-independent periplasmic) transporters, TAT secretion VI (twin-arginine translocation pathway) and Ton-Tol system transporters (Figure 4). ABC transporters were found to be mainly involved in a wide diversity of substrates uptake: amino acid, lipids, sterols or drugs transport (265 proteins) (Wilkens, 2015). On the contrary to specialist bacteria, generalists have evolved a wide diversity of broad-specificity and energy intensive transporters. In this way, *E. mobile* BBCC367 could rapidly and tightly regulate its metabolism and use energetically expensive transporters for nutrient acquisition. TRAP transporters (55 proteins) are widely used by marine bacteria that live in Na^+^ rich environments. TRAP transporters co-transport two Na^+^ ions with one substrate molecule; the energetic cost is thus lower than ABC transporters, while the use of the substrate-binding protein allows the transporter to function with an affinity similar to an ABC transporter (Mulligan et al., 2011). Both ABC and TRAP transporters were found to be abundant in *Roseobacter* clade bacteria compared to other bacterial groups, thus making them more adapted for dilute and heterogeneous growth substrates (Tang et al., 2012). Other transporters such as TAT secretion pathway serve to export proteins across the cytoplasmic membrane (Posey et al., 2006). ABC transporters play a crucial role in metal resistance and detoxification of metals via metal efflux transport (Bruins et al., 2000). Ton-Tol transporters (26 proteins) allow bacteria to take up scarce resources when nutrients are limited in the environments (Tang et al., 2012). Interestingly, it was previously shown, that generalist bacteria had a higher number of secreted proteins compared to specialist bacteria, due to the higher ectoenzymatic activity observed in particle-attached bacteria (mainly generalist) vs. free-living (mainly specialist) bacteria (Lauro et al., 2009).

Heavy metal tolerance to zinc, cobalt, mercury, arsenic, and copper is facilitated by the following proteins in *E. mobile* BBCC367: Zn- Czcd, Cobalt transporter, MerT, Acr3, CueO, and CopA pumps (Anton et al., 1999; Bondarczuk and Piotrowska-Seget, 2013; Hobman and Crossman, 2015; Ladomersky and Petris, 2015). *E. mobile* BBCC367's theoretical proteome harbors 12 proteins involved in copper resistance including a copper translocating protein (CopA), a multi-copper oxidase (CueO) that confers copper tolerance (Cooksey, 1993). Another type of bacterial resistance to toxic compounds includes multidrug efflux pumps including MFS, RND, MATE, and ABC transporters. Toxic compounds removed through this system include acrilavine, beta-lactam, aminoglycoside, fluoroquinone, and cationic drugs (Figure 4).

#### Life in Nutrient-Limited Environments

*E. mobile* BBCC367 has many pathways involved in ammonia, phosphate, and sulfate assimilation (Figure 4) thus making *E. mobile* BBCC367 efficient at utilizing the available nutrients. *E. mobile* BBCC367's theoretical proteome harbors hipA gene involves in the dormancy or quiescence to allow survival in adverse conditions, commonly triggered by a lack of nutrients (Rittershaus et al., 2013). HipA is a kinase, which phosphorylates and inactivates the translation factor EF-Tu (Figure 4, yellow star), facilitating persistence or quiescence (Dawson et al., 2011).

#### Quorum Sensing and Chemotaxis

Generalist bacteria are enriched in genes involved in motility (N), defense mechanisms (V), and signal transduction (T) (Lauro et al., 2009). Quorum sensing (QS) is a density-dependent bacterial communication mechanism that functions by secretion and detection of small signaling molecules called auto-inducers (AIs). In *E. mobile* BBCC367, lipolysis-stimulated lipoprotein receptor (LSR) family proteins facilitate QS (depicted in orange, Figure 4). LsrA, LsrB, LsrC and LsrD (mid right of Figure 4) are all protein compounds, which allow extracellular detection of autoinducers (Hooshangi and Bentley, 2011) whereby the lsrACDB genes encode the ABC transporters allowing the import of autoinducers (Taga et al., 2001). In many Gram-negative bacteria QS is regulated by the LuxI/LuxR type system where LuxR senses the autoinducer (Taga et al., 2003). Different types of autoinducers were identified as being produced and sensed in bacteria (LaSarre and Federle, 2013). Within the *Roseobacter* clade some *Ruegeria* species including *R. atlantica* were shown to produce and detect N-acyl homoserine lactone (AHL) signaling molecules (Mohamed et al., 2008). In *E. mobile* BBCC367, autoinducer 2 (AI-2), another type of signaling molecule, was characterized. AI-2 (Figure 4) facilitates interspecies cell-cell signaling, motility and chemotaxis (Marques et al., 2011). Chemotaxis is a way of bacteria to respond to external stimuli that may either be attractants or repellents. The stimulus is detected via chemoreceptors, which are transmembrane proteins. In *E. mobile* BBCC367 the complex of histidine protein kinase CheA and linker proteins CheW allows chemotactic modulation of flagellar activity depending on environmental stimuli (Wang et al., 2010).

#### DNA Repair Mechanisms

A total of 45 genes were found to be involved in oxidative stress defense and five DNA repair mechanisms: nucleotide excision repair (NER), mismatch repair proteins (MMR), photoenzymatic repair (PER), DNA recombination and genome stabilization (Friedberg, 2003) (Figure 4, red stars indicate DNA repair mechanisms). This suggests that *E. mobile* BBCC367 can cope with a variety of different stresses. Interestingly, this strain was found to be capable of single strand DNA uptake to undergo genetic transformation. DNA uptake can be useful to enhance the genetic diversity (e.g. acquisition of metabolic functions, virulence traits or antibiotic resistance), for DNA repair or as source of carbon, nitrogen, and phosphorus (Chen and Dubnau, 2004).

#### Phage-Related Genes

The *E. mobile* BBCC367's theoretical proteome harbors 25 predicted phage genes including five lysozyme prophages. Viruses represent the largest source of genetic material on the planet, and are likely the major vehicle for gene transfer in the oceans (Duhaime et al., 2011). According to Paul (2008), half of the cultivable marine bacteria contain phage-like particles by prophage induction, allowing the repression host growth in times of resource partitioning. The phage shock protein system (Psp) is responsible for regulating proton motor protein force in conditions exerting stress on the inner membrane of bacteria (Jovanovic et al., 2010). PspA was found to be relevant in response to different conditions (8/16, Table S1) including heavy metal stress.

### Proteomic Investigation of *E. Mobile* BBCC367

A total of 3,083 proteins were classified into three different proteomes according to their protein expression patterns under the 16 different conditions: (i) core proteome (584 proteins) (ii) accessory proteome (2,144 proteins) and (iii) unique proteome (355 proteins). The core proteome is the set of proteins, which were expressed in all 16 conditions and is the minimal set of proteins required to sustain life (Yang et al., 2015). The accessory proteome refers to the situational or adaptive set of proteins, which were expressed under at least 2 conditions. Finally, the unique proteome refers to those proteins, which were expressed in a single condition and are highly situational and specifically adaptive. Most proteins identified in *E. mobile* BBCC367 (the accessory and unique proteomes, 81% of expressed proteome) were subject to change in expression, whereas relatively few proteins were constitutively expressed (core proteome, 19% of the expressed proteome).

#### Core Proteome (584 Proteins)

**Figure 5:**
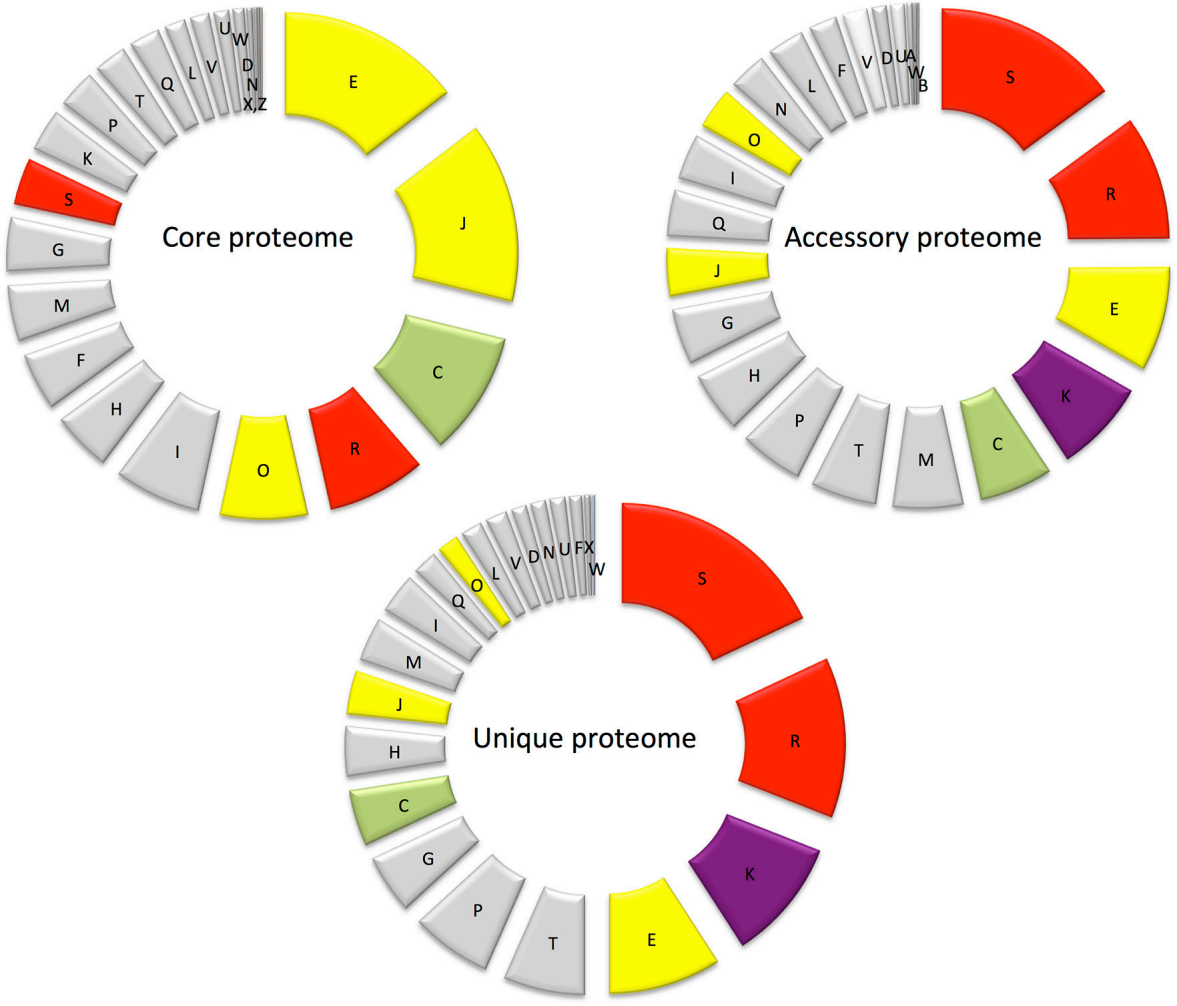
Diagram showing the abundance of proteins in each COG in the core proteome, randomly expressed and unique proteomes.

Major differences between the core and accessory proteomes include the greater prominence of COGs E (amino acid transport and metabolism) and J (Translation, ribosomal structure, and biogenesis) and an under-representation of hypothetical proteins (COG S—function unknown and COG R—general function predicted only) in the core proteome (Figure 5). This indicates the importance of translation/ribosomal proteins and ribonucleases that control the rates of RNA maturation/decay, which influences the rate of encoded protein synthesis (Nicholson, 1999). Furthermore, COG C (energy production and conversion) and COG O (post translational modifications, protein turnover and chaperones) were also more prominent in the core proteome than the accessory proteome (Figure 5). The prominence of COG C facilitates utilization of diverse carbohydrate sources in *E. mobile* BBCC367. Protein chaperones function to prevent protein aggregation and efficient protein folding (Hartl et al., 2011) and play an important role in protecting cells from different stress conditions (Skorko-Glonek et al., 2008). Seven protein chaperones were identified in the core proteome of *E. mobile* BBCC367 (Table S1) including HtrA (EPIB1_1000), chaperone protein DnaK (EPIB1_180), chaperone protein DnaJ (EPIB1_179), heat shock protein 60 family co-chaperone GroES (EPIB1_2287), and heat shock protein 60 family chaperone GroEL (EPIB1_2286). Survival protein SurA precursor (EPIB1_1904) was identified in *E. mobile* BBCC367'*s* core proteome and was found to be involved in correctly folding outer membrane proteins (Lazar and Kolter, 1996).

In *E. mobile* BBCC367'*s* core proteome 54 ATP-dependent transporters (Table S1) were identified, indicating the significance of ATP hydrolysis for transportation of substrates. Eight TRAP transporters were found in the core proteome (Table S1) suggesting they play a crucial role in transporting essential substrates for cell functionality like ABC transporters. Universal stress protein UspA (EPIB1_2652) was also found in the core proteome (Table S1). UspA can be expressed under a large variety of stress conditions such as stationary phase, exposure to heat or metals (Nyström and Neidhardt, 1994; Kvint et al., 2003). This suggests that UspA can be involved in multiple stresses in *E. mobile* BBCC367 (Table S1).

COGs W (extracellular structures), Q (Secondary metabolites biosynthesis, transport, and catabolism) and I (Lipid metabolism) were not represented in the core proteome, but were only found in the accessory and unique proteomes and are thus non-essential, yet adaptive.

#### Accessory Proteome (2,144 Proteins)

**Figure 6:**
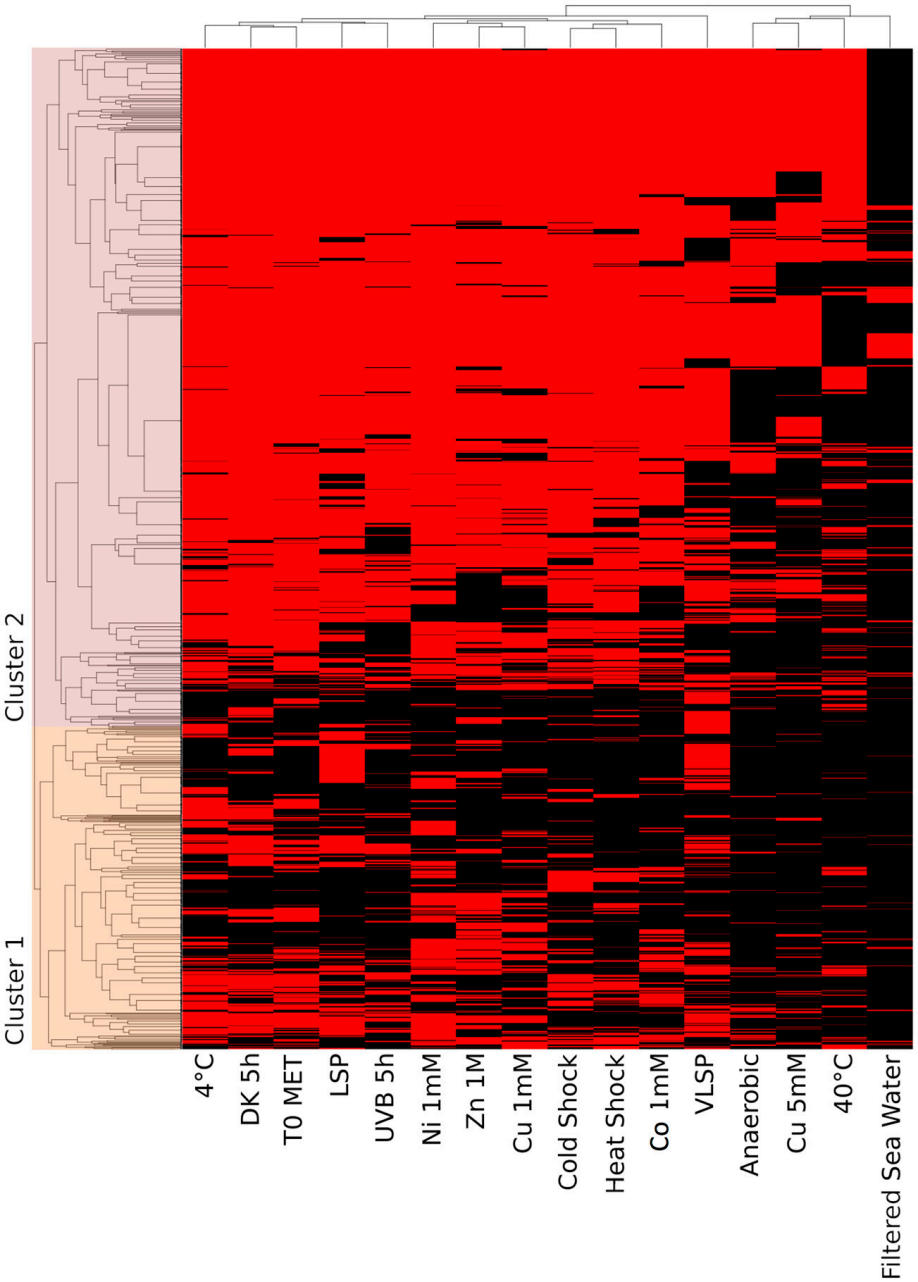
Hierarchical cluster analysis (HCA) of the accessory proteome. The heat map is linked by a dendrogram representing clustering of the different experimental treatments (top), and protein expression profiles (side). Color code: Black: protein not identified/Red: protein identified by mass spectrometry.

**Figure 7:**
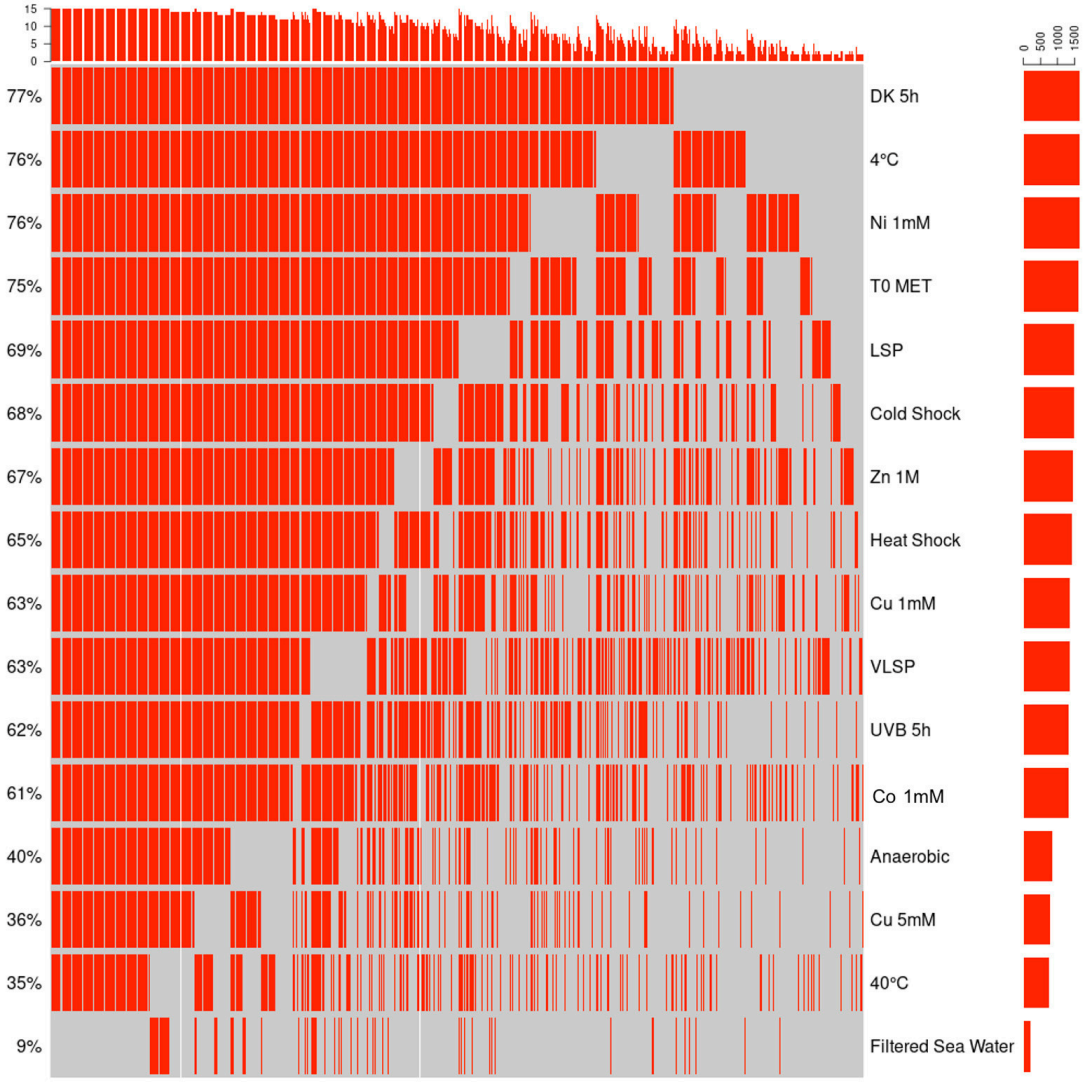
Qualitative visualization (oncoprint) of the accessory proteome. The x-axis represents the identified proteins (red, non-identified proteins in gray), the y-axis the different conditions.

This is the largest of the three identified proteomes in *E. mobile* BBCC367 and comprised proteins whose expression was facultative but could not be linked to dedicated conditions. Still, some non-random patterns could be identified across the 16 tested conditions (Figures 6, 7). We observed two main groups of conditions, showing similarities in terms of protein expression. The four following conditions namely 40°C, anoxic, 5 mM copper and filtered sea water showed significant differences in the pattern of the expressed proteome compared to other conditions (Figure 6). Overall *E. mobile* BBCC367 would repress the expression of many genes under those four conditions that we could qualify as the most stressful conditions. Indeed high temperature, high concentration of copper, lack of oxygen, or low nutrient concentration (filtered seawater) would lead to gene expression being repressed and/or protein degradation. We identified significantly fewer proteins in those four conditions (1,383, 1,438, 1,365, and 786 proteins for the conditions 3, 5, 9, and 11, respectively) in comparison to the other bacterial growth conditions (about 2,000 proteins) (Figure 6 and Table S2).

Regarding the grouping according to the protein function, we observed two main branches that divided two groups of proteins (Clusters 1 & 2, Figure 6). The expression of the proteins from Cluster 1 was found to be more variable from one condition to another and this group of proteins was over-represented by energy related proteins (COG C) and amino acid transporters (COG E). Proteins from Cluster 2 were found to be more constitutively expressed across all conditions. This group was dominated by proteins involved in transcription (COG K) and hypothetical proteins (COG S).

A total of 11 proteins involved in DNA repair were found in the accessory proteome including DNA mismatch repair protein MutS (EPIB1_13), MutL (EPIB1_285), DNA repair protein RecN, (EPIB1_2179) and LexA (EPIB1_1683) with RecA expressed in *E. mobile* BBCC367's core proteome (Table S1). MutS, MutL, RadA and RecN were all expressed in at least 10 conditions with MutS, LexA, and RecN expressed in 15/16 conditions (Table S1). The expression of these proteins in *E. mobile* BBCC367 demonstrate that DNA repair strategies and the SOS response can provide resistance under different stresses, thus maintaining the integrity of the genome in *E. mobile* BBCC367 as early as possible in the DNA damage process.

#### Unique Proteome (355 Proteins)

This is the smallest of the three proteomes defined in *E. mobile* BBCC367. Proteomic investigation of the unique proteome can infer information on particular protein functions associated with specific environmental conditions. COGs S (unknown function) and R (general function) are the largest contributors of the unique proteome followed by COG K (transcription) as shown in Figure 5. Regulation at the level of transcription is less energetically costly than regulation at the level of translation as transcriptional regulation prevents the production of unnecessary proteins. For clarity purposes, the unique proteome obtained for each individual condition will be discussed according to the three main stressor groups: temperature (90 proteins), nutritional limitation (103 proteins), and metals toxicity (162 proteins) (Table S1).

#### Proteins Expressed Under Temperature Related Conditions

A total of 31 proteins involved in transcriptional regulation were identified in the unique proteome of *E. mobile* BBCC367 (S1) (9.2% of the unique proteome). The LysR-type transcriptional regulator is the largest family of transcription regulators in prokaryotes (Santiago et al., 2015), having a regulatory role over genes whose products can be involved in metabolism, cell division, QS, secretion and oxidative stress response (Maddocks and Oysten, 2008). Ten LysR-type proteins were identified in *E. mobile* BBCC367'*s* unique proteome (S1), including: transcriptional regulator, LysR family (EPIB2_971), three of which were expressed in condition 3 (40°C). These transcriptional regulators most likely play a role in tolerating higher temperatures (40°C), by controlling protein expression. Interestingly, several proteins of the flagellum were found exclusively in the temperature-related conditions. Temperature impacts the motility by changing the polarity of the membrane (Lewus and Ford, 1999). In the unique proteome six proteins involved in motility/QS/chemotaxis were identified: flagellar M-ring protein FliF (EPIB1_88), flagellar hook-associated protein FlgK (EPIB1_82) and autoinducer-binding transcriptional regulator LuxR (EPIB2_776). LuxR is an autoinducer-dependent activator of transcription of the lux operon to activate the signal transduction pathway for QS (Qin et al., 2007). The expression of FliF and FlgK in the unique proteome indicates that they play an important role in flagellar motility/function (which may coincide with chemotaxis strategies) under different temperature conditions.

#### Proteins Expressed Under Nutrient Limitation

During starvation (LSP/vLSP condition, Table 1) bacterial cells strive to uptake nutrients from the limited environment and thus, proteins actively involved in the transport of nutirents are key for cell survival (Helloin et al., 2003). We identified a significant predominance of ABC transporters specialized in the transport of oligopeptides, sugar, polyamine, tricarboxylate as well as several TRAP transporters that are less energetically costly. Eleven ABC transporters and three TRAP transporters were identified in *E. mobile* BBCC367'*s* unique proteome as being expressed under conditions 6 and 7 (LSP and vLSP). A range of ABC transporters were expressed including a sugar ABC transporter (EPIB1_641), a histidine ABC transporter (EPIB2_734) and an oligopeptide ABC transporter, the periplasmic oligopeptide-binding protein OppA (PEPIB1_22). This suggests that a wide range of molecules could be transported in conditions 6 and 7 (LSP and vLSP). Other transporters including such as the oligopeptide transport system permease protein OppC and the phosphate homeostasis/uptake protein PhoU (EPIB2_537 and EPIB1_1570, respectively), as well as a few membrane/transmembrane proteins denoted to COG R (general function) were also expressed in conditions 6 and 7. The abundance of transporters and especially ABC transporters was also identified in *R. pomeroyi* in nutrient-poor conditions (Christie-Oleza and Armengaud, 2010). It is therefore suggested that *E. mobile* BBCC367 can withstand nutrient limiting conditions through the expression of different membrane transporter proteins and other general membrane proteins.

#### Proteins Expressed Under Metal Related Conditions

**Figure 8:**
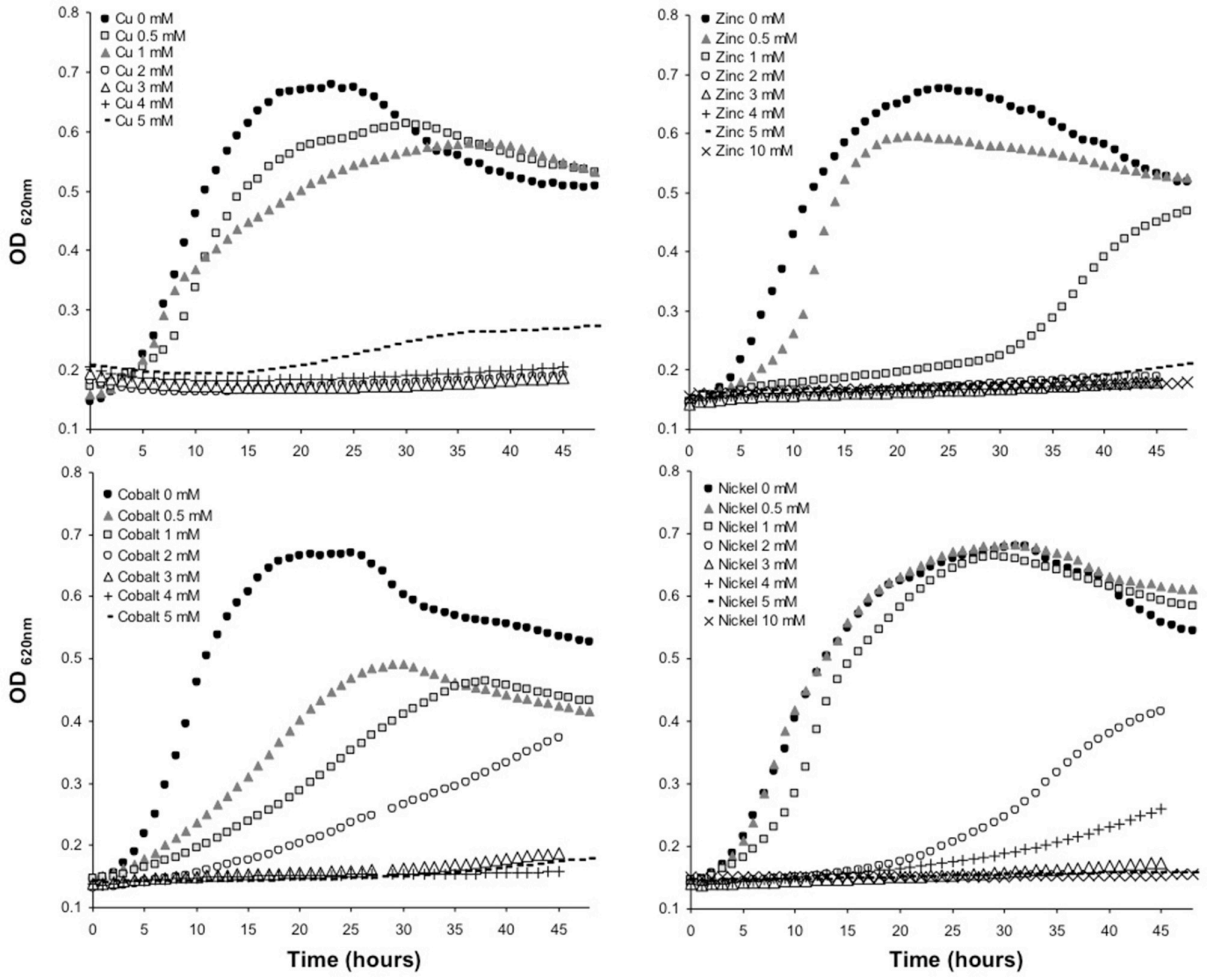
Growth curves of *E. mobile* BBCC367 in presence of different concentration of copper, zinc, nickel and cobalt.

**Figure 9:**
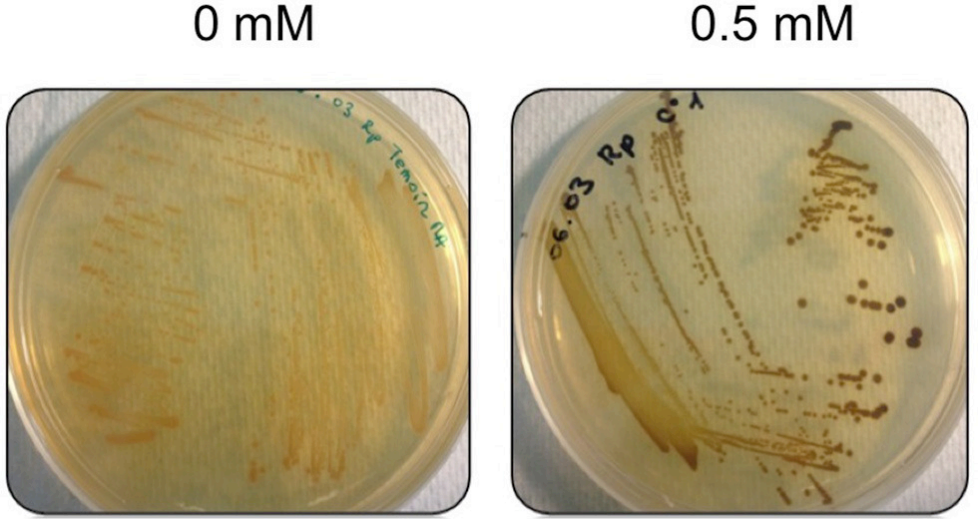
Morphotypes of *E. mobile* BBCC367 in presence of copper.

The minimum inhibitory concentration (MIC) in *E. mobile* BBCC367 for the 4 tested metals was 1 mM for Zinc, 2 mM for Cobalt, 4 mM for Nickel and 5 mM for Copper (Figure 8). In line with those results, the largest proportions of the unique proteomes related to metal stresses were dedicated to nickel, zinc and copper tolerance implying that resistance to nickel, zinc and copper at low concentration (1 mM) demands the greatest physiological change (Table S1). Active transport or efflux systems represent the largest category of metal resistance systems (Nies, 1999; Bruins et al., 2000). In *E. mobile* BBCC367, fourteen ABC transporters were characterized when the cells were grown in presence of metals as well as a mercuric transport protein MerT (PEPIB2_188), twin-arginine translocation protein TatC (EPIB1_1226) and cobalt-zinc-cadmium resistance protein CzcD (PEPIB2_31). CzcD was only expressed in condition 12 (Zn 1mM), which indicates that CzcD has an important role in providing resistance and tolerance to zinc in *E. mobile* BBCC367. Two copper translocating P-type ATPases were found to be expressed in Copper, Zinc, and Nickel (1 mM) conditions. ATPases including P-type ATPases have crucial roles in heavy metal resistance and constitute the basic defense against heavy metal cations (Nies, 2003). Those proteins would play a key role in detoxifying metals via efflux transport. In *E. mobile* BBCC367, the blue copper oxidase CueO precursor (PEPIB2_173) was identified in condition 10 (Cu 1 mM), however not in condition 11 (Cu 5 mM) (Table S1). Higher concentrations of copper cause lipid peroxidation and protein damage (Dupont et al., 2011) and might have altered CueO at 5 mM in *E. mobile* BBCC367. Indeed as presented in Figure 8, bacterial growth was significantly impacted by the highest concentration of copper of 5 mM. Two other multicopper oxidases were expressed in both conditions 10 and 11 (Cu 1 mM and Cu 5 mM) (Table S1) allowing resistance of *E. mobile* BBCC367 to copper. Both copper resistance protein B's and copper homeostasis protein CutE were expressed in both conditions 10 and 11 (Cu 1 mM and Cu 5 mM) (Table S1). Copper chaperone CopZ, a metal chaperone involved in zinc homeostasis was identified in the copper and nickel conditions, respectively and would allow an effective storage of copper ions. Colony forming unit (CFU) on agar plate containing copper showed a change of color from orange to dark brown due to the storage of the copper inside the cells (Figure 9).

Finally, the unique proteome of *E. mobile* BBCC367 showed that transcriptional regulators are key actors in the resistance to metals. 14 transcriptional regulators including LysR-family transcriptional regulators were identified as being expressed under metal stress conditions (Table S1). LysR-family transcriptional regulators were previously identified to be important in cellular survival upon metal treatments (Latorre et al., 2014; Santiago et al., 2015).

### Impact of UVB Radiation on *E. mobile* BBCC367 Assessed by Quantitative Proteomics

By quantitative proteomics, a total of 65 proteins were quantified, out of which 22 were up-regulated (gray), 11 were down-regulated (white) and 32 showed no significant differential regulation (not shown) following UV exposure compared to the dark control (Table 3).

**Table 3:** List of the quantified proteins differentially regulated between the UV stress and Dark control conditions (white: down-regulated proteins/gray: up-regulated proteins).

**Accession number**	**Protein name**	**Ratio UV/DARK**	***p*-value**
EPIB2_549	L-arabinose-binding periplasmic protein precursor AraF (TC 3.A.1.2.2)	0.01	0.034
EPIB1_2796	Ornithine cyclodeaminase (EC 4.3.1.12)	0.02	0.008
EPIB1_242	FIG01073164: heat shock protein HspQ	0.04	0.003
PEPIB1_27	Regulatory protein, GntR:Bacterial regulatory protein, GntR	0.08	0.031
EPIB1_1072	FIG01031704: hypothetical protein	0.13	0.051
PEPIB2_97	Putative protein-S-isoprenylcysteine methyltransferase	0.19	0.024
EPIB1_868	GCN5-related N-acetyltransferase	0.21	0.040
EPIB1_907	Chromosome partition protein smc	0.24	0.037
EPIB1_1054	5-formyltetrahydrofolate cyclo-ligase (EC 6.3.3.2)	0.25	0.042
EPIB1_2841	Xanthine dehydrogenase, iron-sulfur cluster and FAD-binding subunit A (1.17.1.4)	0.30	0.009
EPIB1_40	SAM-dependent methyltransferase, BioC-like	0.41	0.036
EPIB1538	Biotin-protein ligase (EC 6.3.4.15)	2.12	0.059
EPIB1_2370	Hypothetical protein	2.33	0.011
EPIB1_533	L-carnitine dehydratase/bile acid-inducible protein F (EC 2.8.3.16)	2.41	0.012
PEPIB1_117	peptidase, M23/M37 family protein	2.44	0.053
EPIB1_1974	Arginine-tRNA-protein transferase (EC 2.3.2.8)	2.92	0.058
EPIB1_671	2-octaprenyl-6-methoxyphenol hydroxylase (EC 1.14.13.-)	3.17	0.054
EPIB1_715	Quinolinate synthetase (EC 2.5.1.72)	3.84	0.024
EPIB1_1377	NADH-FMN oxidoreductase	4.68	0.051
EPIB1_393	SSU ribosomal protein S11p (S14e)	5.45	0.037
EPIB1_798	HAD-superfamily hydrolase, subfamily IA, variant 1 family protein	5.51	0.012
EPIB1_384	SSU ribosomal protein S5p (S2e)	6.81	0.051
EPIB1_2072	Hypothetical protein	6.96	0.041
EPIB1_1273	Putative cytoplasmic protein	10.36	0.022
EPIB1_1980	DNA-binding response regulator, LuxR family	11.59	0.045
EPIB1_2053	Transcriptional regulator, GntR family	12.73	0.012
EPIB1_1233	FIG01030832: hypothetical protein	14.57	0.060
PEPIB2_172	Blue copper oxidase CueO precursor	18.97	0.024
EPIB2_571	3-hydroxyisobutyrate dehydrogenase (EC 1.1.1.31)	21.68	0.004
EPIB1_1965	bacterial luciferase family protein	31.69	0.005
EPIB1_1479	Transcriptional regulator, GntR family domain/Aspartate aminotransferase (EC 2.6.1.1)	95.51	0.018
EPIB1_1808	OsmC-like family protein	119.46	0.039
EPIB2_300	Hypothetical protein	273.83	0.006

*Fold change ratios and p-values were obtained from triplicates*.

#### Transcription and Translation

Transcription factors belonging to the GntR family were identified as being involved in extreme resistance to gamma radiation, UV radiation and ROS response in *Deinococcus radiodurans* and *Rhodobacter* sp. (Dulermo et al., 2015; Pérez et al., 2017). Three GntR family-associated proteins were quantified in *E. mobile* BBCC367: GntR: bacterial regulatory protein (PEPIB1_27; ratio value 0.08), transcriptional regulator, GntR family (EPIB1_2053; ratio value 12.73) and transcriptional regulator, GntR family domain/aspartate aminotransferase (EPIB1_1479; ratio value 95.51). Although GNAT proteins transferring acetyl groups to a wide variety of substrates (Favrot et al., 2016) are known to be involved in stress response (Xie et al., 2014), the GCN5-related N-acetyltransferase (GNAT) (EPIB1_868) was found to be down-regulated (ratio value 0.21) under UVB radiation (Table 3). Methyltransferases including the putative protein-S-isoprenylcysteine methyltransferase and the BioC-like SAM-dependent methyltransferase were both down-regulated under UVB radiation (Table 3). Methyltransferases are associated with DNA methylation, heterochromatin formation, and repression of DNA synthesis. In the absence of UVR, these methyltransferases were more abundant, potentially being involved in heterochromatin formation of UV-related genes. Finally, ribosomal proteins (EPIB1_393 and EPIB1_384; ratio values 5.45 and 6.81, respectively) were found to be up-regulated upon UVB radiation such as in *Photobacterium angustum* S14 (Matallana-Surget et al., 2012a) and in *Sphingopyxis alaskensis* (Matallana-Surget et al., 2009). Ribosomal proteins play a role in UV resistance in *E. mobile* BBCC367 by maintaining translational/ribosomal stability.

#### Metabolism and Amino Acid Transport

The L-arabinose-binding periplasmic protein precursor AraF (EPIB2_549; ratio value 0.01), and ornithine cyclodeaminase (EPIB1_2796; ratio value 0.02) both involved in amino acid transport pathways, showed the lowest ratio values in *E. mobile* BBCC367 and significantly low ratio values compared to previous UV quantitative proteomic studies (Matallana-Surget and Wattiez, 2013). Proteins involved in amino acid synthesis in *P. angustum* S14 were also down-regulated under UVB treatment (Matallana-Surget et al., 2012a).

The arginine-tRNA-protein transferase exhibited a 2.92 fold change in protein abundance in the UV stress condition compared with the dark control. This protein contributes to protein degradation pathways and thus, may be up-regulated to degrade UV-damaged proteins.

Two proteins involved in folate metabolism (5-formyltetrahydrofolate cyclo-ligase; EPIB1_1054; ratio value 0.25 and xanthine dehydrogenase, iron-sulfur cluster and FAD-binding subunit A; EPIB1_2841; ratio value 0.30) were down-regulated in response to UVB radiation (Table 3), confirming former studies showing UV stressed cells would preferentially use energy for particular UV resistance mechanisms rather than general metabolic processes (Matallana-Surget et al., 2012a; Matallana-Surget and Wattiez, 2013). The oxydoreductase, 3-hydroxyisobutyrate dehydrogenase (EPIB2_571), which carries out valine, leucine and isoleucine degradation and is involved in the production of acetyl-CoA was found to be up-regulated in the dark condition (0.41 UV: dark ratio). Similarly in *Chlorella variabilis*, the 3-hydroxyisobutyrate dehydrogenase was down-regulated in response to UVR stress (Poong et al., 2018). The relatively higher expression of 3-hydroxyisobutyrate dehydrogenase in the dark condition causes a reduction in valine, leucine, and isoleucine, this is known to stimulate protein degradation and inhibit protein synthesis (Freund and Hanani, 2002). Biotin-protein ligase (EPIB1_538; ratio value 2.12) was found to be up-regulated under UVB radiation in *E. mobile* BBCC367 (Table 3). Biotin is an essential co-factor aiding the replenishment of the tricarboxylic acid cycle and amino acid metabolism (Salaemae et al., 2016). Thus, the up-regulated biotin-protein ligase is likely to maintain the functionality of these metabolic processes under UV stress. Quinolinate synthetase (EPIB1_715) also known as NadA in *E. coli* (Ollagnier-de Choudens et al., 2005) was up-regulated (ratio value 3.84) in response to UVB radiation (Table 3). This protein is involved in the NAD biosynthesis pathway and could lead to an increase amount of reduced NADH in *E. mobile* BBCC367, which allows for the pumping of sodium ions and an increase in ATP synthesis (Matallana-Surget et al., 2012a).

#### Proteins Involved in Oxidative Stress Response

The OsmC-like family protein (EPIB1_1808) was found to be up-regulated (ratio value 119.46) in *E. mobile* BBCC367 upon exposure to UVB radiation compared to the dark control (Table 3). The OsmC protein was shown to have a key function in oxidative stress defense (Dubbs and Mongkolsuk, 2007).

Interestingly the blue copper oxidase CueO precursor (PEPIB2_172), involved in periplasmic detoxification of copper, was up-regulated (ratio value 18.97) under UVB radiation compared to the dark control (Table 3). The periplasmic multicopper oxidase CueO was reported to be involved in copper homeostasis and protection against oxidative stress in *E. coli* (Grass et al., 2004). Copper oxidizes catechols, which leads to the production of hydrogen peroxide and hydroxyl radicals, and catechols are able to reduce Cu^2+^ to Cu^+^ (Grass et al., 2004). Overall, these reactions lead to increased levels of ROS and increased intake of toxic Cu^+^. CueO can reduce the amount of Cu^+^ and thus, the accumulation of ROS (Grass et al., 2004). Blue copper oxidase CueO precursor is proposed to play an important role in reducing the amount of oxidative stress by reducing the amount of UV induced ROS in *E. mobile* BBCC367.

## Conclusion

Our proteogenomic analysis allowed us to identify almost 70% of the *E. mobile* BBCC367 theoretical proteome, and revealed that 81% of the proteins were subject to changes in expression depending on growth conditions, whereas 19% were constitutively expressed. *E. mobile* BBCC367 tolerates and resists environmental stress including temperature, heavy metal exposure, nutrient deprivation and UVB radiation. Cell envelope biogenesis proteins were the only specific class of proteins expressed in response to all tested metals, indicating holistic importance of cell envelope biogenesis in metal tolerance. Findings from the late stationary phase revealed amino acid metabolism and transport proteins to be highly important in the limited environment associated with late stationary phase growth. Proteins involved in transcription/translation regulation and amino acid transport were found to be essential to favor the resistance of *E. mobile* BBCC367 under UVR. The diversity of stress responses analyzed in this study provides a valuable platform for future more targeted in-depth studies.

## Author Contributions

SM-S conceived and designed the experiments. SM-S and LI performed the experiments. PNG and MF genome sequencing. SM-S, JW, KL, CR, JM, DG and SIR analyzed the data. SM-S, HT, PL, and RW contributed reagents, materials, and analysis tools. SM-S, CR, JM, and KL wrote the paper. SM-S, JW, HT, and LI proofreading.

### Conflict of Interest Statement

The authors declare that the research was conducted in the absence of any commercial or financial relationships that could be construed as a potential conflict of interest.
