# Supplementary material for: Proteogenomic Analysis of Epibacterium Mobile BBCC367, a Relevant Marine Bacterium Isolated From the South Pacific Ocean
Source: Front Microbiol. 2018 Dec 21;9:3125. doi: 10.3389/fmicb.2018.03125 (PMC6308992; doi:10.3389/fmicb.2018.03125)
Supplement: Supplementary file 2 [file Table_2.docx]

Table S2. Number of identified proteins and percentage of proteome coverage for each condition

| Condition | Number of identified proteins | % of proteome coverage |
| --- | --- | --- |
| 1 | 2,255 | 49.5 |
| 2 | 2,063 | 45.3 |
| 3 | 1,383 | 30.3 |
| 4 | 2,008 | 44.1 |
| 5 | 1,438 | 31.6 |
| 6 | 2,092 | 45.9 |
| 7 | 2,023 | 44.4 |
| 8 | 786 | 17.2 |
| 9 | 2,205 | 48.4 |
| 10 | 1,966 | 43.1 |
| 11 | 1,365 | 30.0 |
| 12 | 2,061 | 45.2 |
| 13 | 1,917 | 42.0 |
| 14 | 2,285 | 50.1 |
| 15 | 1,922 | 42.2 |
| 16 | 2,260 | 49.6 |
